# Graphene Oxide Nano-Concentrators Selectively Modulate RNA Trapping According to Metal Cations in Solution

**DOI:** 10.3389/fbioe.2020.00421

**Published:** 2020-05-25

**Authors:** Valentina Palmieri, Lorena Di Pietro, Giordano Perini, Marta Barba, Ornella Parolini, Marco De Spirito, Wanda Lattanzi, Massimiliano Papi

**Affiliations:** ^1^Dipartimento di Neuroscienze, Università Cattolica del Sacro Cuore, Rome, Italy; ^2^IRCCS Fondazione Policlinico Universitario Agostino Gemelli, Rome, Italy; ^3^Dipartimento Scienze della Vita e Sanità Pubblica, Università Cattolica del Sacro Cuore, Rome, Italy

**Keywords:** graphene, nucleic acid, miRNA, diagnostics, nanotechnology

## Abstract

With recent advances in nanotechnology, graphene nanomaterials are being translated to applications in the fields of biosensing, medicine, and diagnostics, with unprecedented power. Graphene is a carbon allotrope derived from graphite exfoliation made of an extremely thin honeycomb of sp2 hybridized carbons. In comparison with the bulk materials, graphene and its water-soluble derivative graphene oxide have a smaller size suitable for diagnostic platform miniaturization as well as high surface area and consequently loading of a large number of biological probes. In this work, we propose a nanotechnological method for concentrating total RNA solution and/or enriching small RNA molecules. To this aim, we exploited the unique trapping effects of GO nanoflakes in the presence of divalent cations (i.e., calcium and magnesium) that make it flocculate and precipitate, forming complex meshes that are positively charged. Here, we demonstrated that GO traps can concentrate nucleic acids in the presence of divalent cations and that small RNAs can be selectively released from GO-magnesium traps. GO nano-concentrators will allow better analytical performance with samples available in small amounts and will increase the sensitivity of sequencing platforms by short RNA selection.

## Introduction

With advances in next-generation sequencing, the human transcriptome has been deciphered and the regulatory roles of a large fraction of small RNAs suggest that they might represent poorly invasive disease biomarkers in diagnostics (Masud et al., 2019). Liquid biopsy, as an example, investigates cell-free nucleic acids in the peripheral blood of patients and is considered one of the most advanced non-invasive tool for early diagnosis, staging, and prognosis (Finotti et al., 2018). The analysis of circulating free RNAs has been mostly focused on microRNA (miRNA) patterns as epigenetic signatures associated with the neoplastic transformation but also many other diseases (Burgos et al., 2014; Finotti et al., 2018). However, the clinical significance of miRNAs might not be clearly demonstrated without an accurate optimization and standardization of the protocols used for their isolation and assessment (El-Khoury et al., 2016). In order to quantify miRNA levels in plasma or in other patient fluids, several types of nanotechnologies have been proposed (Finotti et al., 2018). However, cell-free nucleic acid measurement approaches suffer from important drawbacks, such as the instability of small RNAs and their low concentration (e.g., femtomolar to picomolar levels) and complicate tagging and isolation procedures (El-Khoury et al., 2016; Finotti et al., 2018). Even if new methods based on miniaturization and nanomaterials have been exploited to improve current detection limits (Chen et al., 2018) the development of a sensitive and reliable assay for miRNA enrichment is still lacking.

In recent years, the carbon allotrope graphene has become extremely popular in the biomedical field, due to its unique interactions with cells and organic compounds (Dhinakaran et al., 2020). Graphene is a bi-dimensional material made of a honeycomb of carbon atoms and available in sizes ranging from few nanometers (graphene quantum dots) to large flakes of several microns in lateral size. Among the many graphene derivatives, the soluble oxidized form graphene oxide (GO) is the most studied for biomedical applications, as it interacts with cells, proteins, and bacteria in unique ways (Dreyer et al., 2014; Palmieri et al., 2018a, b).

Both GO and its reduced by-product rGO have a high affinity for single-stranded nucleic acids (ssNAs) via hydrogen bonds and/or π-π interactions, especially for short ssNAs (i.e., <20 nucleotides in length), given the slower diffusivity of longer ssNAs (Liu et al., 2016; Yan et al., 2017). This observation led to the development of GO-based methods for NA extraction from complex media (Hashemi et al., 2014). However, some restrictions in the use of GO and its derivatives are still limiting their widespread use.

Graphene oxide binding to nucleic acids is based on the interaction with backbone phosphate, and this could lead to indiscriminate reactions with either double-stranded nucleic acids (dsNAs) or ssNAs, and to reduced sensitivity in distinguishing the dsNAs to the ssNAs. For this reason, rGO has been preferred, given its direct interaction with nucleotide residues exposed in ssNAs like miRNAs (Yan et al., 2017). On the other hand, rGO, compared with GO, is difficult to handle because it precipitates in complex biological fluids. Recent studies indeed proposed to functionalize rGO with magnetic beads (Yan et al., 2017) or gold nanoparticles (Bao et al., 2019) to avoid this limitation. rGO samples can be obtained from GO using several alternative reduction protocols, which causes high variability in oxygen content, stability, and size (Dreyer et al., 2014; Palmieri et al., 2019) and could lead to poor reproducibility of results from different laboratories. Finally, there is a lack of a standardized method for efficient desorption of adsorbed NAs from both GO and rGO surface (Liu et al., 2016). This step is crucial for the subsequent analysis of the isolated RNA samples.

In this work, we propose a nanotechnological method for enriching total RNA or selectively small RNA molecules (<100 nucleotides in length as miRNAs) in solution. To this aim, we developed a protocol based on the unique trapping effects of GO nanoflakes in the presence of divalent cations. Indeed, cations like calcium and magnesium quickly adsorb on GO surface. After adsorption, GO flocculates and precipitates forming complex structures with size and stability largely dependent on the type and concentration of the ion involved (Wu et al., 2013; Yang et al., 2016). This has been also named the “trapping effect of GO,” when it occurred in the presence of bacteria that remain encapsulated and insulated in large GO traps (Ji et al., 2016; Palmieri et al., 2017a, b; De Maio et al., 2019). Here, we demonstrate that the trapping occurs also with nucleic acids and that can be reversed selectively at high pH to selectively release and concentrate small RNAs. Conversely, large RNAs remain on GO surface, especially in the presence of Mg^2+^, which is known to form RNA clamps in cells (Petrov et al., 2011). This method is simple and specific and does not involve any complex preparation process, multistep probe functionalization, or tagging. GO nano-concentrators of small RNAs will give the opportunity for refining the analytical performance of existing platforms by reducing the saturation of purification columns by large RNA species (including the large amount of mRNAs and rRNAs), increasing the sensitivity of sequencing platforms and will allow short RNA selection even in samples available in small amounts such as cerebrospinal fluids (Kopkova et al., 2018) and tears (Pieragostino et al., 2015).

## Materials and Methods

### GO Characterization

Graphene oxide (4 mg/ml) was purchased from GrapheneA (Spain) and diluted in MilliQ RNA and DNA free water for further experiments. GO size and thickness were evaluated by atomic force microscopy (AFM) (Nanowizard II JPK Instruments AG, Berlin, Germany) as previously reported (Bugli et al., 2018). GO hydrodynamic properties and surface charge were measured by dynamic light scattering (DLS) and zeta potential analysis by means of Zetasizer Nano ZS (Malvern, Herrenberg, Germany) as previously reported (Palmieri et al., 2019). The lateral size of samples was calculated using intensity particle size distribution peaks as reported previously (Lotya et al., 2013). Samples have been prepared using a fixed GO concentration (2 mg/ml) and a final concentration of cations in solution of 5, 1, or 0.5 mM adding CaCl_2_ or MgCl_2_. After the interaction, samples were diluted to avoid multiple scattering previous DLS measurements.

### Nucleic Acids Extraction

Genomic DNA (gDNA) and total RNA samples were alternatively extracted from U87 human glioblastoma cells (purchased from ATTC). Cells were maintained in Dulbecco’s modified Eagle’s medium (Sigma-Aldrich) supplemented with 10% fetal bovine serum (FBS, EuroClone), 2% penicillin–streptomycin (Sigma-Aldrich), and 2% L-glutamine (Sigma-Aldrich). gDNA was extracted using E.Z.N.A.^®^ SQ Blood DNA Kit (Omega BioTek), according to the manufacturer’s instructions. Total RNA samples were collected by TRIzol reagent method (Invitrogen), using the standard protocol (Di Pietro et al., 2017; Barba et al., 2018). gDNA and RNA purity and concentration were assessed using a UV spectrophotometer (DU 800, Beckman Coulter).

### GO Interaction With Genomic DNA (gDNA) and Total RNA

Ten microliters of GO (at initial concentration of 2 mg/ml) have been added to 1 μl of ddH_2_O, CaCl_2_, or MgCl_2_. After 10 min, 9 μl of gDNA or total RNA (60 ng/μl) have been added to the solution. The final concentration of cations in solution was 5, 1, or 0.5 mM. A synthetic miRNA (cel-miR-39-3p, mature miRNA sequence: UCACCGGGUGUAAAUCAGCUUG, ThermoFisher) was added to RNA samples, to a final concentration of 30 pM, to be assessed as an exogenous control for small RNAs for qPCR analysis. In particular, the use of this exogenous miRNA allowed overcoming the possible issue deriving from the different expression levels of any endogenous cellular miRNAs among biological samples. Samples were incubated for 15 min and then centrifuged for 10 min at 14,000 × *g*. Nucleic acid concentration was measured in the supernatant of samples by measuring absorbance at 260 nm with Cytation 3 Take 3 reader (BioTek, United States) or fluorescence peak at 520 nm when 5′6-FAM-labeled ssNAs were used in the experiment. In particular, a 22-nucleotide-long DNA sequence (ACTGCGGCTTAGGTTAGCATTG) was randomly designed, considering a sequence with the same length and CG and AT content of a typical miRNA, and purchased by IDT Integrated DNA technologies with a 5′-56 FAM modification. The presence and integrity of total RNA in the supernatant of samples have been additionally evaluated by agarose gel electrophoresis in all the experimental conditions.

### Real-Time PCR

The levels of *GAPDH* (*glyceraldehyde-3-phosphate dehydrogenase*) transcripts and of cel-miR-39-3p in samples were evaluated using reverse transcription quantitative real-time PCR (qPCR). More in detail, the same quantity of each sample was reverse transcribed using GoScript Reverse Kit (Promega) and *GAPDH* transcript levels were subsequently amplified with StepOne system (Applied Biosystems), using Syber Green master mix (GoTaq qPCR Master Mix Kit, Promega) and specific primer pairs (forward primer: AACTTTGGTATCGTGGAAGGA; reverse primer: GGCAGTGATGGCATGGAC) (Barba et al., 2012; Di Pietro et al., 2017). In parallel, total RNAs were also used as templates for miRNA reverse transcription, using TaqMan^TM^ Advanced miRNA cDNA Synthesis Kit (ThermoFisher), according to the manufacturer’s instructions. cel-miR-39-3p levels were amplified using TaqMan^TM^ Fast Advanced Master Mix (ThermoFisher) and a specific TaqMan^TM^ Advanced miRNA Assay (Assay ID#478293_mir, ThermoFisher).

## Results

### GO Loses Stability in the Presence of Divalent Cations

**FIGURE 1:**
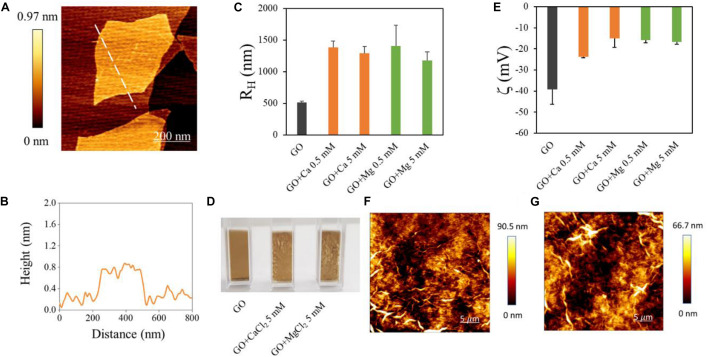
Characterization of GO samples used and of GO interaction with divalent cations. **(A)** AFM representative image of GO flake and a height profile obtained along the dashed line **(B)**. Hydrodynamic radius of GO samples in different solutions obtained by DLS **(C)**. Photographs of cuvettes containing GO in different solutions **(D)**. Zeta potential of GO in different solutions **(E)**. Representative images of GO aggregates in Ca^2+^ **(F)** or Mg^2+^ **(G)** obtained by AFM.

Graphene oxide nanoflakes used in our experiments had a mean lateral size of ∼700 nm as measured from AFM imaging (Figure 1A). AFM was used to measure the average GO flakes thickness, which was ∼0.8 nm (see a representative line profile of a GO flake in Figure 1B). The size of GO flakes was confirmed by DLS, used to analyze hydrodynamic properties of samples (Figure 1C). Data represent the peak of size intensity distributions reported in Supplementary Figure S1, together with the lateral size of each sample obtained by DLS (Supplementary Table S1). It is known that GO is stable in water but rapidly aggregates in the presence of divalent cations. This effect was exploited in environmental applications envisaging water purification (Hegab and Zou, 2015) and was reported to trap biological entities such as bacteria and fungi (Palmieri et al., 2017a, b). In order to induce RNA trapping, we have chosen Ca^2+^ and Mg^2+^ ions among cations known to change GO properties in solution mainly because heavy metal cations (Cr^3+^, Pb^2+^, Cu^2+^, Cd^2+^, and Ag^+^) destabilize too strongly GO (Yang et al., 2016). Ca^2+^ and Mg^2+^ have similar hydration shells (Yang et al., 2016) and the same positive charge, so that a reduced repulsion between RNA phosphates and GO surface can be expected, though Mg^2+^ is known to be most effective in stabilizing RNA in folded structures (Draper, 2004).

From a macroscopic point of view, GO and cations quickly formed a dense cloud in solution as soon as they were mixed (Figure 1D). Representative AFM images of aggregates are shown in Figure 1E. The hydrodynamic radius of GO was measured after the addition of Ca^2+^ or Mg^2+^ at two concentrations (0.5 and 5 mM). The addition of divalent cations caused an increase of R_H_ up to ∼1400 nm from ∼500 nm as reported in Figure 1C. From a negative value of ∼−40 mV of bare GO, the surface zeta potential (Figure 1E) increased with Ca^2+^ and Mg^2+^, confirming a positive charging of GO surface in the presence of cations. GO meshes created with cations can be separated from the rest of the solution by precipitation or more quickly with centrifugation; this avoids functionalization with magnetic beads or other nanoparticles for further analysis. Representative images of GO aggregates in the presence of Ca^2+^ (Figure 1F) and Mg^2+^ (Figure 1G) obtained by AFM.

### Bare GO Has Low Affinity for Nucleic Acids, but Divalent Cations Dramatically Increase Adsorption of DNA and RNA on GO

**FIGURE 2:**
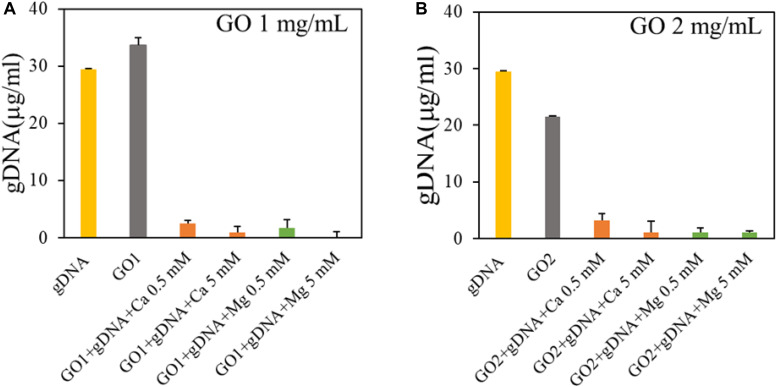
Analysis of binding of GO with genomic DNA in the presence of GO at 1 mg/ml **(A)** or GO at 2 mg/ml **(B)**. Concentration of DNA in supernatants is reported after UV spectroscopic analysis.

We evaluated GO interaction with single-strand and double-strand NAs. First, we measured GO affinity for dsDNA by incubating GO with gDNA at a final concentration of 30 ng/μl. gDNA concentration was measured in the supernatant fractions recovered after incubation with different GO samples and centrifugation, and compared to the control sample (gDNA with buffer; Figure 2).

Our results showed that GO (1 mg/ml) does not adsorb gDNA (Figure 2A), only if the concentration of GO is doubled, 40 μg of GO adsorb ∼0.15 μg of gDNA. As expected the interaction between GO and dsDNA is not favored, since the binding between GO and nucleic acids is largely dependent on hydrogen bonds and/or π-π interactions with nucleobases and nucleobases are occluded inside the double helix in gDNA (Liu et al., 2016). Further, repulsive interactions occur between phosphate backbones and GO negative surface. With the addition of divalent salts, the gDNA is completely adsorbed on GO even at very low salt concentration (0.5 mM). This can be explained by the bridging of cations between GO and the dsDNA phosphate backbone both charged negatively (Wu et al., 2011). With Ca^2+^/Mg^2+^ in solution, the concentration of gDNA adsorbed is ∼5 times higher (0.58 μg of gDNA per 40 μg of GO) without significant differences between the cations.

**FIGURE 3:**
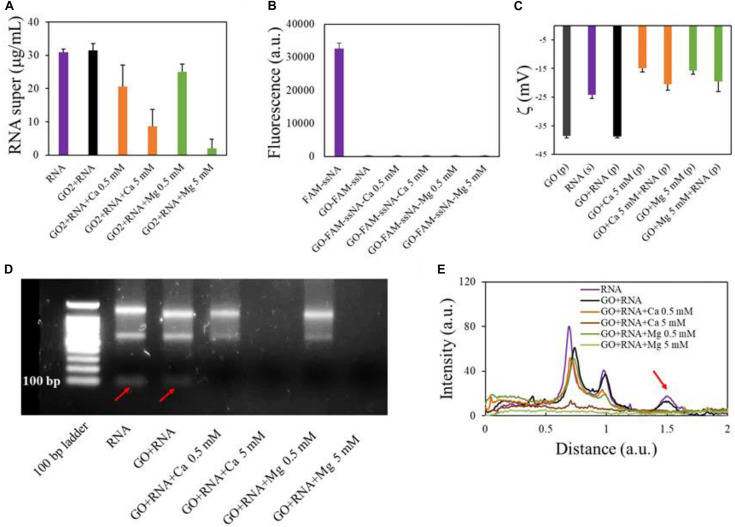
Analysis of GO binding with total RNA. Concentration of RNA in supernatants after interaction with GO or GO and cations measured by UV spectroscopy **(A)**. Quantification of fluorescent FAM-ssNA in supernatants after interaction with GO **(B)**. Zeta potential of GO samples after RNA adsorption in different conditions **(C)**. Electrophoretic analysis of supernatants after interaction with GO **(D)** and line profile analysis of gels **(E)** with red arrow highlighting small RNA peak (corresponding to the band under 100 bases).

We then analyzed the interaction of GO with total RNA extracted from cells in culture (U87 glioblastoma cells). First, we incubated GO (2 mg/ml) with total RNA (final concentration 30 ng/μl) added with the synthetic cel-miR-39-3p as an exogenous control for small RNA (final concentration 15 pM) (Figure 3A). As observed with gDNA, GO alone has poor affinity for total RNA, which is adsorbed only if a solution with high Ca^2+^ or Mg^2+^ concentration is added to GO prior to the incubation with RNA. Twenty micrograms of GO adsorbed 0.4 and 0.55 μg of total RNA with 5 mM Ca^2+^ and 5 mM Mg^2+^, respectively. This result might seem in contrast with reports from literature that have shown, even if in low amounts, adsorption of RNA even from bare GO (Wu et al., 2011; Yan et al., 2017). We wanted to reproduce conditions of previous studies and we used, instead of total RNA, the FAM-ssNA, as a model NA. We incubated FAM-ssNA (final concentration, 30 ng/μl) with different GO samples and then measured fluorescence from supernatants. We observed that FAM-labeled ssNA is readily adsorbed also by GO (Figure 3B). This led us to hypothesize that studying adsorption of FAM-tagged nucleic acids might be biased by the strong interaction of FAM probe with GO surface.

Analysis of surface zeta potential of GO pellets after RNA adsorption confirmed that RNA molecules were deposited on GO surface (Figure 3C) only in the presence of divalent cations. Indeed, zeta potential of GO pellets after incubation with RNA was equal to GO alone. Conversely, after incubation, pellets of GO-Ca^2+^-RNA and GO-Mg^2+^-RNA had surface charge substantially different from GO-Ca^2+^ and GO-Mg^2+^ pellets, respectively.

Electrophoresis analysis of supernatants confirmed the UV spectroscopy results (Figure 3D) and in particular displayed that smaller RNAs (<100 nucleotides in length) were better adsorbed since they were not visible in the supernatant even in samples with small cation concentrations as shown by the disappearance of the low-molecular-weight peak in the line profiles of electrophoretic gels (red arrow in Figure 3E). This is confirmed by results in literature that reported that longer RNAs bind to GO with slower diffusivity (Liu et al., 2016).

**FIGURE 4:**
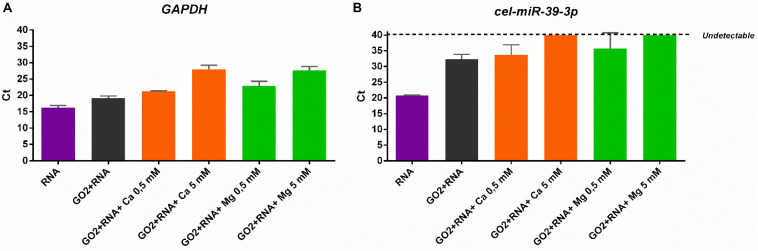
**(A)** qPCR analysis of GAPDH and **(B)** cel-miR-39-3p levels in supernatants after incubation with GO and different cations.

To better clarify the higher affinity of GO for smaller RNAs in the presence of divalent cations, qPCR analysis was performed on supernatant samples recovered after incubation of RNA samples with GO. Considering that higher Ct (cycle threshold) values correspond to a lower amount of transcript levels in the sample, we have compared the different samples amplifying the same volume of each supernatant. Our data showed that longer RNAs are significantly adsorbed more at high concentration of cations, as demonstrated by the increased Ct for *GAPDH* transcript amplification (one-way ANOVA, *p* < 0.0001; Figure 4A). In particular, qPCR analysis indicated that both Ca^2+^ and Mg^2+^ had the same ability to retain RNA. Instead, miRNAs started binding even if only GO is present (Figure 4B). Very interestingly, in the presence of the high concentration (5 mM) of both Ca^2+^ or Mg^2+^, GO was able to adsorb the total amount of small RNAs, since cel-miR-39 became undetectable in the supernatant fraction after incubation (one-way ANOVA, *p* < 0.0001; Figure 4B).

Altogether, these data demonstrate efficient trapping of total RNA in solution by GO-divalent cations with faster recognition of small RNAs.

### Small miRNAs Are Selectively Desorbed After the Increase of pH From GO Flakes

It is important to release small RNAs bound to GO surface for further PCR/sequencing analysis. Indeed, at the critical concentration of 4 μg/ml, PCR reactions are inhibited by the GO in solution (Wang et al., 2017). The desorption of NAs from GO is indeed a complex problem (Liu et al., 2016). Many studies in the literature have reported that the addition of either cDNA, random DNA, or albumin could detach RNA from GO surfaces as well as the use of solutions of isopropanol or base that could facilitate the specific desorption of miRNAs (Hashemi et al., 2014). We have tried several buffers and incubation conditions reported in the literature but without success (data not shown). This is because the bond between RNA and GO cations has indeed proven to be very robust. The desorption of RNA was successfully reached only after two washes with H_2_O and with incubation of 5 min with 1.5 mM Tris buffer (pH 8.8) at room temperature. These conditions were chosen based on literature data that indicate that the adsorption is favored at lower pH and low temperature (Wu et al., 2011). Further, at high pH, the degree of deprotonation of the carboxyl groups at the edges can induce more stability of GO sheets and less precipitation and aggregation (Wu et al., 2013).

**FIGURE 5:**
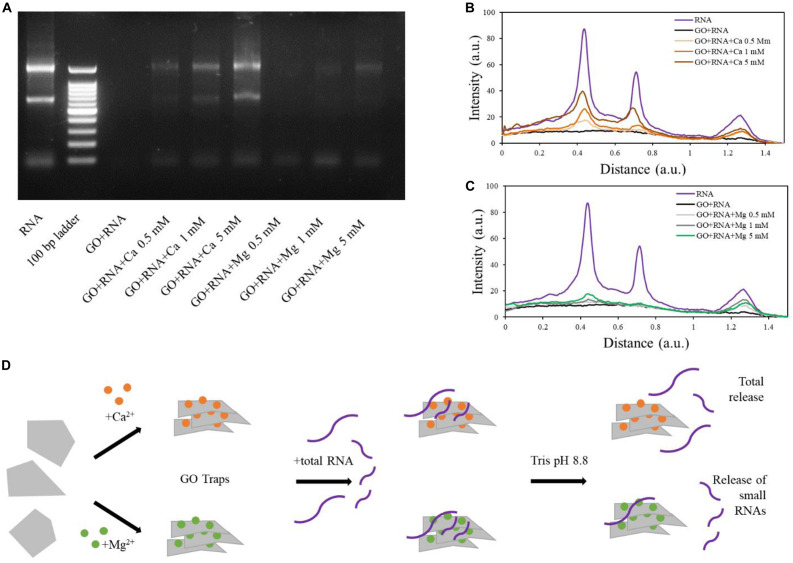
Electrophoretic analysis of supernatants after release from GO sheets in Tris buffer at high pH **(A)**. Line profile analysis of electrophoresis gel and comparison of RNA and GO-RNA samples with GO-calcium **(B)** and GO-magnesium **(C)**. Schematic illustration of our results **(D)**.

In tris buffer, the RNA was successfully released from GO-Ca^2+^samples (both small and long RNAs; Figure 5). The quantity of released RNA increased in a concentration-dependent manner, reaching the higher desorption levels in 5 mM samples, in line with the amount of RNA adsorbed on GO (Figure 5A). Interestingly, the desorption from GO-Mg^2+^ with our buffer affected only smaller RNAs and was not concentration-dependent, whereas the longer RNAs remained “trapped” and there is a small release of large RNA only at high Mg^2+^ concentration (see line profiles in Figures 5B,C). The GO-RNA sample did not display any signal on the electrophoretic gel as visible in Figure 5A, since the RNA did not bind to GO and was totally recovered after the first centrifugation (see Figure 3C).

## Discussion

With recent advances in nanotechnology, nanomaterials are being translated to applications in the fields of biosensing, medicine, and diagnostics, with unprecedented power (Chen et al., 2018). In this paper, we exploit the unique surface features of the bidimensional GO and its trapping ability in the presence of calcium/magnesium ions to create a method to selectively concentrate total RNA/small RNAs in solution. The main advantage of using GO compared to other nanoparticles for nucleic acid-related application is its surface-to-volume ratio. One-dimensional nanomaterials indeed possess high surface-to-volume ratio, which provides large active surface area for the interactions with nucleic acids, and this strongly favors the adsorption of molecules and ultimately leads to better sensitivity (Varghese et al., 2015). Graphene derivatives have also exceptional thermal, optical, and electronic properties that allow easy building of sensor devices. We demonstrated the feasibility of applying GO-based nanotechnologies for a highly sensitive separation of RNA species based on size. In particular, we demonstrated that GO can enrich small RNAs from complex solutions of RNA and how to easily detach ssNAs from GO surface. We indeed used a two-step protocol based on GO trapping of molecules, mediated by divalent cations (calcium and magnesium) and without specific probes. GO is first decorated with divalent ions and then incubated with total RNA. When in solution with RNA, GO adsorbs nucleic acid molecules with high efficiency thanks to the bridges between phosphate backbone and negative charges of GO surface created with calcium or magnesium ions. GO trapping can completely remove RNA from the solution and concentrate it. After nano-concentration, small RNAs can be selectively released from surface by desorption induced at high pH in the presence of Mg^2+^ ions. Conversely, with calcium, all RNA species are released (Figure 5D). Indeed, magnesium special geometric and energetic relationship with the phosphates of RNA makes tighter packing with large RNA compared to calcium ions. We hypothesize that these results can be explained by a double effect occurring in solution. On one hand, at high cation concentrations (5 mM) that are above the critical coagulation concentration of GO, the large RNA competes for binding to GO surfaces with GO itself, and large RNA is released. On the other hand, the Mg^2+^ ability to make bidentate RNA clamps with large RNAs markedly influenced released studies. Indeed, it has been demonstrated that magnesium, due to its size and charge density, binds more intimately than calcium to the oxyanions of RNA, so that magnesium clamps are made stable by electrostatic interactions, charge transfer, polarization, and exchange interactions (Petrov et al., 2011). This “physiological” role of magnesium is also reflected in our binding results and makes it a selective absorbent of large RNA species. In addition, GO trapping of RNA molecules (>100 nucleotides in length) can also be exploited to improve further transcriptome sequencing that requires sufficient mRNA yield, usually obtained by either poly-A selection or depletion of ribosomal RNA (rRNA) (Choy et al., 2015; Fowler et al., 2018). rRNA indeed constitutes about 80% of the total RNA species in eukaryotic cells, while poly[A]+ mRNA constitutes only about 5%. The use of ribosomal depleted RNA has been shown to recover more information about protein-coding genes, non-coding RNAs, snRNAs, snoRNAs, and repeat elements enabling novel transcribed loci detection (Choy et al., 2015). Our GO-Mg^2+^ based selection represents a fast, reliable method for rRNA depletion. Our results also suggested that GO-Ca^2+^ complexes can instead represent a suitable method to isolate and concentrate total RNA, hence providing a tool for the simultaneous isolation and analysis of both small and large RNA species. This study can pave the way toward the development of novel methods to improve liquid biopsy performance and may contribute to developing innovative approaches for miRNA and/or total RNA detection (Fiammengo, 2017).

## Data Availability Statement

The datasets generated for this study are available on request to the corresponding author.

## Author Contributions

VP, LD, and MB performed experiments and contributed to manuscript writing. VP and MP designed the experiments and methodology and revised the manuscript. GP characterized the graphene samples. WL, OP, and MD contributed to manuscript writing and revision. All authors have read and agreed to the published version of the manuscript.

## Conflict of Interest

The authors declare that the research was conducted in the absence of any commercial or financial relationships that could be construed as a potential conflict of interest.
