# Supplementary material for: Graphene Oxide Nano-Concentrators Selectively Modulate RNA Trapping According to Metal Cations in Solution
Source: Front Bioeng Biotechnol. 2020 May 25;8:421. doi: 10.3389/fbioe.2020.00421 (PMC7261913; doi:10.3389/fbioe.2020.00421)

Supplementary Information

Graphene Oxide Nano-concentrators Selectively Modulate RNA Trapping According to Metal Cations in Solution

Valentina Palmieri*[1,2#], Lorena Di Pietro*[3], Giordano Perini[1,2], Marta Barba[3,2], Ornella Parolini[3,2], Marco De Spirito[1,2], Wanda Lattanzi[3,2], Massimiliano Papi[1,2#]

[1] Dipartimento di Neuroscienze, Università Cattolica del Sacro Cuore, Roma, Italy
[2] Fondazione Policlinico Universitario A. Gemelli IRCSS, Roma, Italy
[3] Dipartimento di Scienze della Vita e Sanità Pubblica, Università Cattolica del Sacro Cuore, Roma, Italy

* These authors contributed equally

# Corresponding author: Massimiliano.papi@unicatt.it, valentina.palmieri@unicatt.it

Table S1 Lateral Size (LS) of GO samples obtained by peak of intensity size distribution according to Lotya et al. 2013 compared to AFM data

Sample	R_H (nm)	LS (nm)
GO (AFM)	R=350±80	700±160
GO	515±19	818±45
GO+Ca 0.5 mM	1386±100	3612±390
GO+Ca 5 mM	1296±104	3266±395
GO+Mg 0.5 mM	1407±242	3694±950
GO+Mg 5 mM	1181±136	2840±490

Figure S1 Representative intensity size distributions of GO samples obtained by DLS

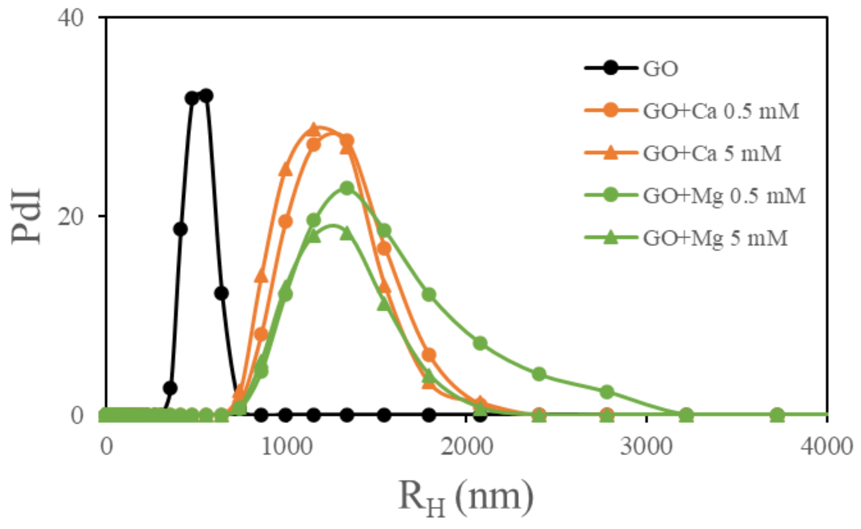

Supplement: Supplementary file 1 [file Data_Sheet_1.pdf]
